# Towards Effective Antiviral Oral Therapy: Development of a Novel Self-Double Emulsifying Drug Delivery System for Improved Zanamivir Intestinal Permeability

**DOI:** 10.3390/pharmaceutics15102518

**Published:** 2023-10-23

**Authors:** Sapir Ifrah, Arik Dahan, Nir Debotton

**Affiliations:** 1 Department of Clinical Pharmacology, School of Pharmacy, Faculty of Health Sciences, Ben-Gurion University of the Negev, Beer-Sheva 84105, Israel; sapirgar@post.bgu.ac.il; 2 Department of Chemical Engineering, Shenkar College of Engineering and Design, Ramat-Gan 52526, Israel

**Keywords:** oral drug absorption, bioavailability, intestinal permeability, microemulsion, nanoemulsion, self-double emulsifying drug delivery system, zanamivir

## Abstract

Self-double emulsifying drug delivery systems have the potential to enhance the intestinal permeability of drugs classified under the Biopharmaceutics Classification System (BCS) class III. One such example is the antiviral agent zanamivir, exhibiting suboptimal oral absorption (with a bioavailability range of 1–5%). To address this challenge, we have developed an innovative oral formulation for zanamivir: a self-double nanoemulsifying Winsor delivery system (SDNE-WDS) consisting of the microemulsion, which subsequently yields final double nanoemulsion (W_1_/O/W_2_) upon interaction with water. Two distinct formulations were prepared: SDNE-WDS1, classified as a W/O microemulsion, and SDNE-WDS2, discovered to be a bicontinuous microemulsion. The inner microemulsions displayed a consistent radius of gyration, with an average size of 35.1 ± 2.1 nm. Following self-emulsification, the resultant zanamivir-loaded nanoemulsion droplets for zSDNE-WDS1 and zSDNE-WDS2 measured 542.1 ± 36.1 and 174.4 ± 3.4 nm, respectively. Both types of emulsions demonstrated the ability to enhance the transport of zanamivir across a parallel artificial membrane. Additionally, in situ rat intestinal perfusion studies involving drug-loaded SDNE-WDSs revealed a significantly increased permeability of zanamivir through the small intestinal wall. Notably, both SDNE-WDS formulations exhibited effective permeability (P*_eff_*) values that were 3.5–5.5-fold higher than those of the low/high permeability boundary marker metoprolol. This research emphasizes the success of SDNE-WDSs in overcoming intestinal permeability barriers and enabling the effective oral administration of zanamivir. These findings hold promise for advancing the development of efficacious oral administration of BCS class III drugs.

## 1. Introduction

Nearly half of the antiviral drugs are classified under the biopharmaceutical classification system (BCS) as class III [1]. Active agents falling into this classification are characterized by high solubility but low permeability across the intestinal lumen, resulting in poor bioavailability [2]. Prominent antiviral drugs in BCS class III include abacavir, didanosine, maraviroc, and zidovudine, approved for the treatment of HIV (Human Immunodeficiency Virus). Additionally, ganciclovir and valganciclovir are prescribed for HCMV (human cytomegalovirus) infections, while trifluridine inhibits viral infections caused by HSV (herpes simplex virus) [3]. HBV (hepatitis B virus) and HCV (hepatitis C virus) are treated with adefovir dipivoxil and sofosbuvir, respectively. Furthermore, some of these agents are utilized in clinical practice to treat multiple human infectious diseases. For instance, lamivudine and tenofovir are indicated for the medical treatment of HIV and HBV. Acyclovir, valacyclovir, and vidarabine are used to treat various members of the herpesviridae family, including HSV and VZV (Varicella–Zoster Virus) [3]. It is not surprising that viruses replicating through similar mechanisms (RNA, DNA, or retroviruses) are often susceptible to the same antiviral agents. For example, ribavirin efficiently inhibits HCV and RSV (Respiratory Syncytial Virus), both of which are RNA viruses. Consequently, with the emergence of new viral infections, such as Severe Acute Respiratory Syndrome Coronavirus 2 (SARS-CoV-2), some of these medications may prove effective if the viruses share similar replicating mechanisms. Regardless of the mechanism of action, improving the oral absorption of these antiviral drugs enhances compliance and hinders the further spread of viral diseases.

Influenza virus is one of the major viral diseases that can erupt into epidemics or even pandemics. In the United States, the average number of influenza patients over the past five years was 32 million, with an approximate 0.1% death rate. The appearance of SARS-CoV-2 led to a decrease in influenza virus incidence during the 2021–2022 season. However, in the subsequent 2022–2023 season, the number of infected individuals increased dramatically by 4.5-fold compared to the previous season [4].

The first-line treatment for the influenza virus typically involves the use of neuraminidase inhibitors, which target and inhibit the neuraminidase enzyme found on the viral envelope. By employing this mechanism of action, neuraminidase inhibitors effectively impede the release of new virions from infected cells, leading to a reduction in the severity and duration of influenza symptoms. Neuraminidase inhibitors, such as oseltamivir, peramivir, zanamivir, and laninamivir octanoate (currently only approved in Japan), are highly effective and widely used for the treatment of both influenza A and influenza B viruses [5]. However, all these drugs (except oseltamivir) fall under BCS class III, indicating challenges in their oral administration. Accordingly, they are administered through inhalation or intravenous routes [6,7,8,9]. Nevertheless, such non-oral administration methods can negatively impact patient compliance and increase the overall cost of the treatment. To address these limitations, it becomes crucial to enhance the intestinal permeability of peramivir, zanamivir, and laninamivir octanoate.

Zanamivir (MW 332.31 g/mol; Log P 4.13), the first discovered neuraminidase inhibitor, has demonstrated higher resistance to influenza mutations compared to oseltamivir [10]. This can be attributed to its hydrophilic nature, which is a result of the presence of a guanidino group and its structural similarity to sialic acid. The unique molecular structure of zanamivir facilitates stronger interactions with the active site of the influenza virus, thereby increasing its overall effectiveness [11]. Currently, zanamivir is administered via inhalation, with an absorption rate ranging from 4% to 17% [12]. Common side effects associated with zanamivir usage include sinusitis and dizziness, while more severe effects, such as bronchospasm (which can be fatal), allergic reactions, and neuropsychiatric events, are particularly observed in children [13,14]. By increasing its ability to be absorbed through the gastrointestinal (GI) tract, oral administration of zanamivir could become a viable option, offering a more convenient and cost-effective treatment approach for influenza.

Numerous drug-delivery emulsion systems have been developed to improve the bioavailability of drugs through the GI tract [15]. These systems not only improve drug solubility but also provide protection and prolong the stability of non-chemically stable drugs [16]. Upon reaching the target tissue, drug activity is enhanced while minimizing side effects. Additionally, drug-delivery emulsion systems have the potential to reduce treatment duration and frequency, which is particularly important in antiviral therapies that may require prolonged or high-dose regimens, resulting in increased treatment costs [17]. Moreover, since emulsions typically consist of an oil phase and an aqueous phase, they can be formulated and customized to accommodate both hydrophilic and lipophilic active substances [18]. Among these systems, oil-in-water (O/W) emulsions are the most commonly utilized in oral administration [19,20,21]. O/W emulsion systems are specifically beneficial for improving the solubility of drugs classified as Biopharmaceutics Classification System (BCS) class II (low solubility, high permeability) and class IV (low solubility, low permeability) [21]. On the other hand, double emulsion systems can augment the permeability of drugs classified as BCS class III by dissolving the drug in the inner aqueous phase and increasing its lipophilicity through the oil phase. Nonetheless, double emulsion systems may suffer from thermodynamic instability over time, limiting their practical applications [22].

In our previous laboratory research, we investigated double emulsion systems encapsulating the active ingredient zanamivir [10]. In this present study, we developed a novel self-emulsifying double emulsion system that holds promise for enhanced intestinal permeability and ultimately improved effectiveness.

Self-double emulsifying drug delivery systems (SDEDDS) are assembled using two distinct primary emulsion types: water-in-oil (W_1_/O) or oil-in-water (O_1_/W). These primary emulsions are combined with an external continuous phase to produce final double emulsions, resulting in water-in-oil-in-water (W_1_/O/W_2_) and oil-in-oil-in-water (O_1_/W/O_2_) configurations, respectively. The former is the prevalent choice for oral administration since the external phase of the W_1_/O/W_2_ system consists of water. In this type of double emulsion, small aqueous droplets are encapsulated within oil droplets within the continuous water phase. The incorporation of hydrophilic emulgators within the oil phase of the primary W_1_/O emulsions is responsible for enabling the self-emulsification process within these delivery systems. Consequently, when the primary emulsion encounters the aqueous environment under GI motility, it self-emulsifies into the final W_1_/O/W_2_ double emulsion structure [23,24]. Considering the inherent instability of double emulsions, the utilization of SDEDDSs, which undergo spontaneous emulsification facilitated by GI movements, proves to be a more effective approach. This is mainly owing to the superior stability achieved by the primary emulsions, transforming SDEDDS into an ideal choice for the delivery of BCS class III drugs [22,25,26,27]. Furthermore, the size of the final droplets of the emulsion systems is a critical factor that significantly influences their stability. Hence, the deliberate reduction in the final SDEDDS following self-emulsification to approximate the dimensions of a nanoemulsion becomes an intriguing strategy. This approach holds the potential to enhance not only the stability but also the permeability of zanamivir, potentially facilitating its absorption and bioavailability within the GI tract.

Microemulsions are a specialized class of emulsions characterized by droplet sizes typically ranging from 5 to 50 nm. These unique emulsions classically comprise water, oil, surfactants, and co-surfactants, while the specific composition and ratios of these components play a crucial role in determining their formation and stability [28]. Microemulsions exhibit distinct advantages, including thermodynamic stability and isotropic clarity [29]. Importantly, these emulsions possess the ability to spontaneously form without the need for vigorous mechanical energy processes such as sonication or homogenization, a feature not shared by nanoemulsions or macroemulsions [30].

Gordon Winsor introduced a classification system for microemulsions, categorizing them into four distinct types [31]: Type I microemulsion is characterized as an O/W microemulsion, where a portion of the oil component is solubilized by the emulsifier, and it achieves equilibrium with an excess of the oil phase. In contrast, Type II microemulsion is classified as a W/O microemulsion, where a fragment of the water component is solubilized by the emulsifier, and it reaches equilibrium with an excess of the aqueous phase. Type III microemulsion is unique in that it involves the solubilization of both oil and water constituents by the emulator. This type of emulsion is commonly named a bicontinuous microemulsion since it maintains an equilibrium state with an excess of the oil and water phases. Winsor Type IV is specifically referring to a single-phase micellar solution [28,32].

Our hypothesis employs the utilization of SDEDDSs, which involve the formulation of primary (W_1_/O) microemulsions that transform into final W_1_/O/W_2_ double emulsions upon mixing with water. The incorporation of hydrophilic emulsifiers within the oil phase of the primary microemulsions facilitates the self-emulsification process within these delivery systems. Consequently, when these microemulsions encounter aqueous environments during GI motility, they culminate in the formation of final double nanoemulsions. This novel class of SDEDDS has been termed Self-Double Nanoemulsifying Winsor Delivery System or SDNE-WDS. Our research focuses on exploring the potential of SDNE-WDSs to enhance the intestinal permeability of the antiviral drug zanamivir through oral administration.

## 2. Materials and Methods

### 2.1. Materials

All organic solvents were of HPLC grade and acquired from Carlo Erba (Milan, Italy). Polysorbate 80, 9,10-diphenyl anthracene, and sorbitan laurate were purchased from Merck KGaA (Darmstadt, Germany). Sucrose stearate was obtained from Sisterna (St. Paul, MN, USA), and zanamivir was obtained from Glentham Life Sciences (Corsham, UK). Rhodamine B, sodium lauryl sulfate (SLS), sodium phosphate dibasic dihydrate, sodium phosphate monobasic dehydrate, and uranyl acetate were acquired from Sigma Aldrich (St. Louis, MO, USA). Carboxymethyl cellulose (CMC) and mineral oil were purchased from Ziv Chemicals Ltd. (Holon, Israel).

### 2.2. Methods

#### 2.2.1. Preparation of SDNE-WDSs with or without Drug Cargo

**Scheme 1:**
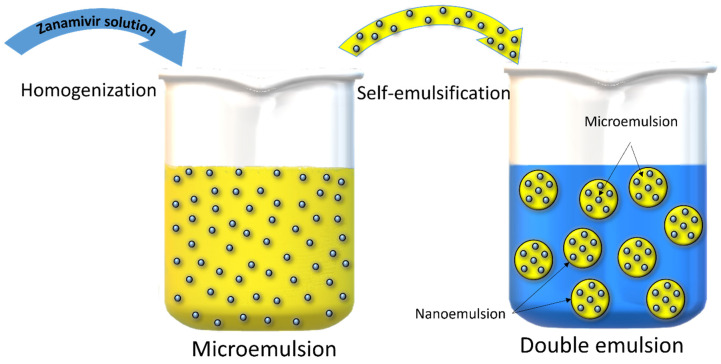
Illustration depicting the preparation process of drug-loaded SDNE-WDSs.

Two formulations were selected based on appropriate material ratios for creating SDNE-WDSs. The oil phase of the formulations was comprised of mineral oil and various surfactants, while the internal aqueous phase (W_1_) contained carboxymethyl cellulose (CMC) as a gelling agent. The difference in the emulsions lies in the utilization of distinct emulgator systems: SDNE-WDS1 employed sorbitan laurate, whereas SDNE-WDS2 contained sucrose stearate. In the context of drug-loaded dispersed systems, a zanamivir solution was incorporated into the W_1_ phase, yielding zanamivir-loaded systems referred to as zSDNE-WDSs. Following homogenization, the resulting primary W_1_/O emulsions were rapidly cooled in an ice bath to room temperature. The self-emulsification process was carried out using a modified technique that was described in prior publications [33,34]. In brief, the final SDNE-WDSs were attained by adding 1 part of W_1_/O emulsions to 6.67 parts of the continuous aqueous phases W_2_, followed by stirring over 30 min at 1000 RPM (as depicted in Scheme 1).

#### 2.2.2. Visualization by Electron Microscope

The morphological examinations of SDNE-WDS1 and SDNE-WDS2 were conducted using transmission electron microscopy (TEM) (Thermo Fisher Scientific (FEI) Talos F200C transmission electron microscope operating at 200 kV, San Jose, CA, USA). A droplet of the specimen was deposited onto a copper grid coated with a carbon film, facilitating a thin film on the grid’s surface. Subsequently, this film underwent negative staining through exposure to a 2% (*w*/*v*) uranyl acetate solution. After drying at ambient temperature, micrographs were captured using a Ceta 16M CMOS camera (Costa Mesa, CA, USA).

#### 2.2.3. Confocal Laser Scanning Microscope (CLSM) Imaging

To visualize the inner aqueous phase in comparison to the outer aqueous phase, the hydrophilic fluorescent probe rhodamine B was dissolved in the W_1_ of the emulsions as a probe for the inner aqueous droplets. Additionally, 9,10-Diphenyl anthracene (DPhA) was dissolved in the oil phase as a fluorescent marker for the oil phase. Fluorescent CLSM images of the double-labeled SDNE-WDSs were obtained using the Zeiss LSM META microscope (Jena, Germany), with rhodamine B parameters set at λ_ex_ 553 nm and λ_em_ 580 nm and DPhA parameters set at λ_ex_ 373 nm and λ_em_ 426 nm.

#### 2.2.4. Analysis of Mean Droplet Size and Surface Charge

The mean droplet sizes and zeta potentials of freshly prepared W_1_/O/W_2_ emulsions derived from SDNE-WDSs were ascertained employing a dynamic light scattering instrument (Zetasizer Nano ZS by Malvern, UK). The particle size distribution of the double emulsions was evaluated subsequent to dilution with water.

#### 2.2.5. Small-Angle X-ray Scattering (SAXS)

SAXS patterns of the mean particle size of the inner aqueous droplets in both SDNE-WDSs were obtained using a SAXSLAB GANESHA 300-XL instrument (Skovlunde, Denmark). The specimen preparation method was carried out according to a procedure detailed elsewhere [35]. The radius of gyration (R_g_) was calculated from the Guinier plot using Equation (1).

(1) $$\ln\left(I_{0}\right)=\ln\left(I\right)-\frac{1}{3}q^{2}{R_{g}}^{2}$$

where I₀ represents the initial scattering intensity, I is the scattering intensity depending on the scatter vector (q) in cm⁻¹, and R_g_ is the radius of gyration in nm.

#### 2.2.6. Determination of Winsor Emulsion Type Using Conductivity Investigation

We utilized conductivity measurements to determine the type of Winsor emulsion of the SDNE-WDS formulations. We employed the ExStik^®^ EC500 instrument (Waltham, MA, USA), which features a stainless-steel electrode optimized for measurements across a wide conductivity range, from 0 to 2000 µs/cm. Additionally, this measurement was performed at a controlled temperature of 25 °C. To identify the type of microemulsion present in SDNE-WDS2, we introduced various concentrations of water into the formulations. A change in the slope of the conductivity measurement of each preparation was indicative of a transition in the microemulsion type, shifting from W/O to bicontinuous and subsequently to O/W [36].

#### 2.2.7. High-Pressure Liquid Chromatography (HPLC) Analysis of Zanamivir

The analysis of zanamivir was conducted using High-Pressure Liquid Chromatography (HPLC) on a Waters 2695 HPLC system (Alliance, Milford, MA, USA). A Hypersil^®^ BDS C18 column with dimensions of 150 mm × 4.6 mm and a particle size of 5 μm was used for chromatographic separation. Data analysis was performed using the Empower Pro software (EMP 2 Feature release 5, Built 2154).

A gradient elution method was applied to identify zanamivir, involving a transition from 10% acetonitrile (*v*/*v*) to 90% acetonitrile (*v*/*v*) in water over a 10-minute period. The flow rate was maintained at 0.5 mL/min, and 10 μL of the sample was injected for analysis. Zanamivir concentration was quantified at a wavelength of 242 nm using a UV detector. This HPLC analysis provided an accurate determination of zanamivir concentration [8].

#### 2.2.8. Quantification of Encapsulation Efficiency

To calculate the entrapment efficiency (EE) of zanamivir within SDNE-WDSs, we employed an ultrafiltration technique. Following the self-emulsification process, 300 µL of the resulting double emulsions were mixed with 300 µL of a 10% SLS solution. Subsequently, these double emulsions were subjected to centrifugation at 14,000 RPM and 40 °C for 15 min to completely disrupt the formulations. The clarified solutions obtained post-centrifugation were subjected to HPLC analysis to quantify the quantity of free zanamivir. The calculation of entrapment efficiency involved determining the percentage (*w*/*w*) of the encapsulated drug relative to the total zanamivir content within the SDNE-WDSs, as per Equation (2) [37,38]. This entrapment efficiency assessment was conducted in triplicate for robust and accurate results.

(2) $$EE\left[\%\right]=\frac{W\;initial\;amount\;of\;drug-W\;untrapped\;free\;drug}{W\;initial\;amount\;of\;drug}\times 100\%$$

#### 2.2.9. In Vitro Release Studies

Experiments evaluating the invitro release profiles of the self-emulsified SDNE-WDSs in comparison to the zanamivir solution were conducted in an aqueous buffer adjusted to match the pH of the small intestine. These experiments utilized dialysis bags with a molecular weight cut-off ranging from 12–14 kDa (Sigma Aldrich, St. Louis, MO, USA). Dialysis techniques are commonly employed for the examination of molecule release from a dispersed drug system due to their ability to isolate the droplets, enabling the active moiety to diffuse into the release medium through a membrane that does not restrict its passage. Accordingly, the pores of the membrane should possess a considerably greater size than that of the released substance. Next, 15 mL of the zanamivir-loaded SDNE-WDSs were placed within the dialysis bag and introduced into 200 mL of phosphate buffer solution (PBS) at pH 6.8, ensuring the presence of a significantly higher volume of media compared to the saturation point at which dissolution would slow (commonly referred to as “sink conditions”). Under continuous magnetic stirring, we extracted 150 µL samples from the dialysis bag at predetermined time intervals (0, 0.33, 0.67, 1, 2, 3, and 4 h). Subsequently, the drug concentration within these samples was determined using an HPLC method after disrupting the SDNE-WDSs [38].

#### 2.2.10. Parallel Artificial Membrane Permeability Assay (PAMPA) for Assessing Passive Diffusion of Encapsulated Zanamivir

The evaluation of the in vitro passive diffusion of encapsulated zanamivir across artificial membranes was carried out using a Pre-coated PAMPA (BD Gentest™, San Jose, CA, USA) [39]. The donor wells were loaded with 300 µL of the respective test groups, which included the free drug, zSDNE-WDS1, and zSDNE-WDS2. The resulting PAMPA sandwich configuration was incubated at 25 °C, during which zanamivir concentrations in both the donor and acceptor plates were quantified using the HPLC technique. The calculation of the effective permeability coefficients, denoted as P*_app_*, was performed according to Equation (3), which is adapted from the Corning^®^ (Tewksbury, MA, USA) Gentest guidebook:

(3) $$P_{app}=\frac{-ln[1-C_{A}(t)/C_{equilibrium}]}{A\times (1/V_{D}+1/V_{A})\times T}$$

where:

$C_{D}\left(t\right)$ represents the compound concentration in the donor well at time *t* [mM]; $C_{A}\left(t\right)$ denotes the compound concentration in the acceptor well at time *t* [mM]; $V_{D}$ represents the volume of the donor well; $V_{A}$ represents the volume of the acceptor well; $C_{equilibrium}$ is calculated as $\left[C_{D}\left(t\right)\times V_{D}+C_{A}\left(t\right)\times V_{A}\right]/\left(V_{D}+V_{A}\right)$; A signifies the filter area; T is the incubation time.

#### 2.2.11. In Situ Single-Pass Intestinal Perfusion (SPIP) Studies

To assess the effective permeability coefficients of zanamivir-loaded zSDNE-WDSs within the proximal jejunal segment, we employed the in situ single-pass rat intestinal perfusion model. All animal procedures adhered to the guidelines set by the Ben-Gurion University of the Negev Animal Use and Care Committee (Protocol IL-30-04-2019). Male Sprague Dawley rats, approximately 300 g in weight (Harlan, Israel), were utilized in this study and were housed and managed in accordance with the Ben-Gurion University of the Negev Unit for Laboratory Animal Medicine Guidelines. The rats underwent an overnight fast (lasting 12–18 h) while having access to water. Random assignments to different experimental groups were produced for each animal. The procedure closely followed the established protocol, as was previously reported [8,39,40]. Briefly, the rats were anesthetized via intramuscular injection with 100 mg/kg of ketamine and 5 mg/kg of xylazine. A midline abdominal incision, approximately 3 cm in length, was made. Permeability measurements were focused on an 11 cm proximal jejunal segment, commencing 2 cm below the ligament of Treitz. The intestinal segment was cannulated at both ends, with an initial perfusion consisting of normal saline solution at 37 °C to establish a steady state, followed by subsequent perfusion involving the collection of samples at 8-min intervals. All perfusion solutions underwent incubation in a water bath at 37 °C and were subsequently pumped through the intestinal segment. Samples from the perfusate were immediately subjected to HPLC analysis. Upon the conclusion of the experiment, the exact length of each perfused jejunal segment was measured. The net water flux during the single-pass rat jejunal perfusion investigations, signifying water absorption within the intestinal segment, was calculated utilizing Equation (4).

(4) $${C^{\prime }}_{out}=C_{out}\times \frac{V_{out}}{V_{outT}}$$

where $C_{out}$ is the drug concentration experimental value in the outlet sample, while the calculated ${C^{\prime }}_{out}$ represents the corrected concentration of the drug. $V_{out}$ is the volume of the outlet sample, and $V_{outT}$ is the theoretical volume that was expected to emerge. The actual absorption rate coefficient across the rat gut wall in the SPIP studies was calculated using Equation (5).

(5) $$P_{eff}=\frac{-Qln({C^{\prime }}_{out}/C_{in})}{2\pi RL}$$

where Q signifies the perfusion buffer flow rate and ${C^{\prime }}_{out}/C_{in}$ denotes the ratio of the outlet concentration (as calculated by Equation (4)) to the inlet concentration of the test drug. R represents the radius of the intestinal segment (set at 0.2 cm), and L signifies the length of the intestinal segment.

#### 2.2.12. Data and Statistical Analysis

Data are presented as means ± standard deviation (means ± SD) or means ± standard error (means ± SE). Statistical analysis to determine significant differences among the experimental groups involved a one-way ANOVA test, followed by Tukey’s test for comparisons between all groups. For pairwise comparisons between the two groups, an unpaired *t*-test was conducted. Significance was established at *p* < 0.05.

## 3. Results

### 3.1. Preparation and Characterization of Stable Zanamivir-Loaded SDNE-WDSs

Numerous formulations were developed to determine the optimal experimental conditions for creating stable SDNE-WDSs. In an attempt to ensure the future feasibility of this delivery system, consideration was given to selecting components from materials already approved for oral administration. The composition of both the oil phase and the gelling agent were similar to those employed in double emulsions previously developed in our laboratory [10]. The choice of hydrophobic emulsifiers, specifically sucrose stearate and sorbitan laurate, was predicated on their demonstrated ability to construct stable double emulsions [41,42]. Furthermore, owing to its favorable physicochemical properties enabling incorporation into the oil phase, polysorbate 80 was the obvious selection as the hydrophilic self-emulsifying agent. Ultimately, two formulations exhibiting robust stability were selected: SDNE-WDS1 and 2. Both primary systems initially existed as microemulsions and, upon dilution with water, transformed into nanoemulsions.

**Figure 1:**
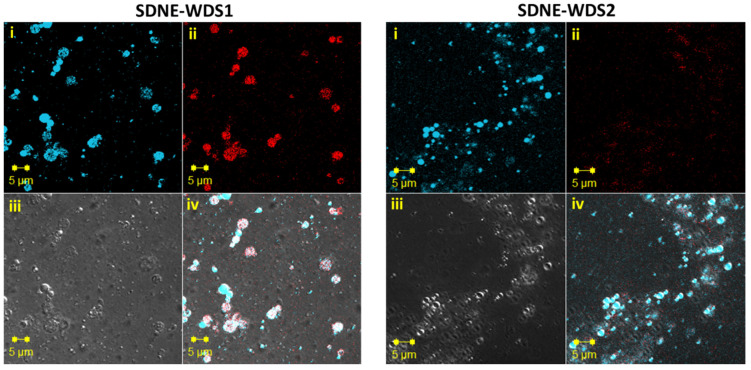
Confocal Laser Scanning Microscopy images of W_1_/O/W_2_ emulsions following self-emulsification of SDNE-WDS1 and SDNE-WDS2: (i) Blue marker DPhA in the oil phase; (ii) Red probe rhodamine B in W_1_; (iii) Optical microscopy images; (iv) Superimposition of images (i,ii), illustrating merged fluorescent signals (blue and red). Magnification ×63.

The CLSM images presented in Figure 1 provide a visual representation of freshly prepared SDNE-WDSs following spontaneous emulsification. Figure 1 (i) represents the inner W1 phase (blue channel), while Figure 1 (ii) depicts the oil phase of the nanoemulsions (red channel). The superimposed images in Figure 1 (iv) demonstrate the co-localization of the red and blue fluorescent signals, signifying the presence of inner aqueous droplets within the oil phase. Moreover, there was no significant detection of red or blue fluorescent signals in the external aqueous phase W2, underscoring the effective encapsulation of the hydrophilic cargo within the inner aqueous phase of the oil droplets.

**Figure 2:**
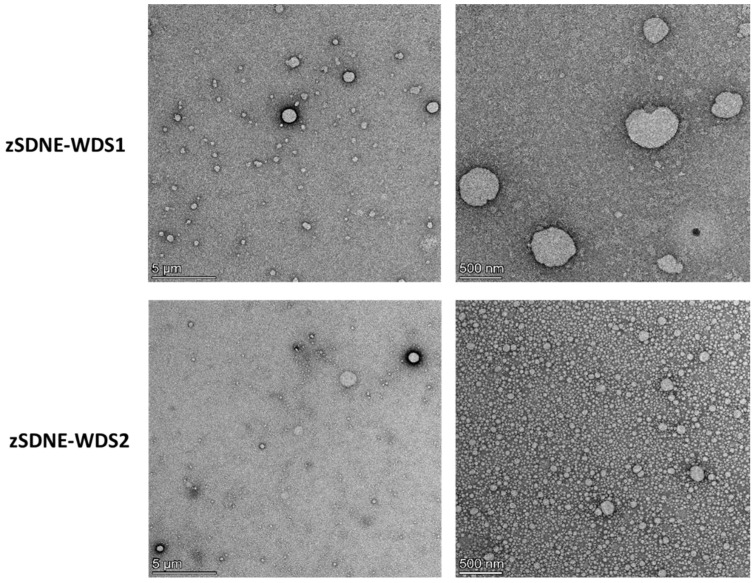
TEM images of W_1_/O/W_2_ emulsions following self-emulsification of zSDNE-WDS1 and zSDNE-WDS2. Specimens were negatively stained using a 2% uranyl acetate solution.

Predominantly, SDNE-WDS1 yielded larger final nanodroplets compared to SDNE-WDS2. This difference in droplet size can be attributed to the distinct oil phase emulsifiers employed, as outlined above. The mean diameters of the W_1_/O/W_2_ droplets were 542.1 ± 36.1 nm and 623.9 ± 53.2 nm for SDNE-WDS1 with and without zanamivir, respectively. For SDNE-WDS2, the mean droplet size was 174.4 ± 3.4 nm, while that of zSDNE-WDS2 was 170 ± 2.3 nm. Remarkably, the presence of zanamivir in the inner aqueous phases of the systems did not result in a substantial difference in the size of the final double emulsion droplets. Additionally, the mean diameter measurements of the SDNE-WDSs aligned with the findings in Figure 2.

Both formulations exhibited a notable negative surface charge. Particularly, the zeta potential of SDNE-WDS1 was −43.9 ± 19.5 mV, while that of zSDNE-WDS1 was −56.93 ± 1.26 mV. For SDNE-WDS2, the zeta potential values were −62.43 ± 0.75 mV and −68.3 ± 17.5 mV, with and without zanamivir, respectively.

It is worth highlighting that comparable drug encapsulation efficiencies were achieved for the resultant diluted double emulsions despite their differences in droplet sizes and zeta potentials. In particular, the encapsulation efficiencies for zSDNE-WDS1 and zSDNE-WDS2 were found to be 77.12 ± 3.98% and 75.66 ± 3.79%, respectively.

Figure 2 depicts the TEM images of zSDNE-WDS1 and zSDNE-WDS2 subsequent to their emulsification, forming W_1_/O/W_2_ double nanoemulsions. Markedly, the outer oil droplets in the W_1_/O/W_2_ structure of zSDNE-WDS2 appeared visibly smaller compared to those in zSDNE-WDS1. Upon closer examination in higher magnification micrographs, zSDNE-WDS2 exhibited a more concentrated presence of smaller droplets, which might be attributed to its mean droplet size. These observations suggest that the choice of formulation and particularly emulsifying agents can influence the size and distribution of droplets within these drug carriers, stressing the importance of fine-tuning these parameters for specific drug delivery applications.

**Figure 3:**
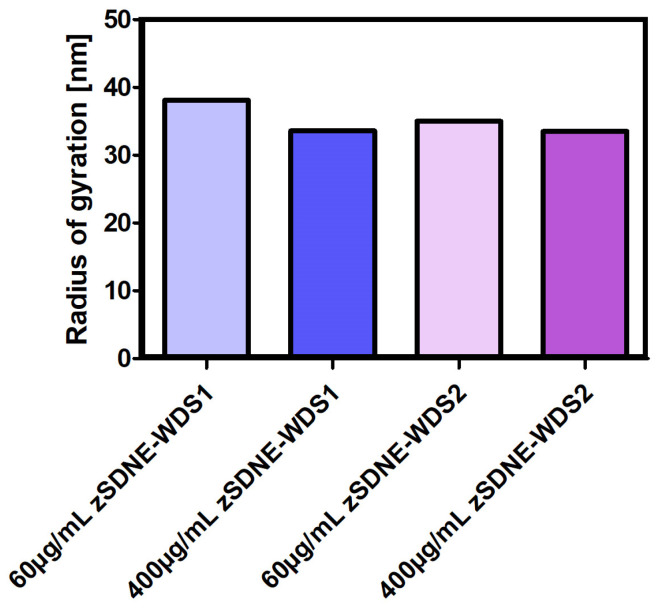
Radii of gyration of the inner aqueous droplets of SDNE-WDS1 and SDNE-WDS2 comprised varying zanamivir concentrations.

The average size of the inner aqueous phases within both Winsor systems was assessed by means of SAXS, as illustrated in Figure 3. Impressively, all the inner microemulsions exhibited a consistent radius of gyration, measuring at an average droplet size of 35.1 ± 2.1 nm. The size of these aqueous droplets remained distinctly stable, showing no considerable variation regardless of the type of oil phase emulsifier employed. Furthermore, the averaged radii of the microemulsions prepared with 60 µg/mL of active agent were comparable to those with zanamivir concentrations exceeding six-fold. This observation implies that the dosage of the active agent can be modified while preserving the structural integrity of the delivery system.

**Figure 4:**
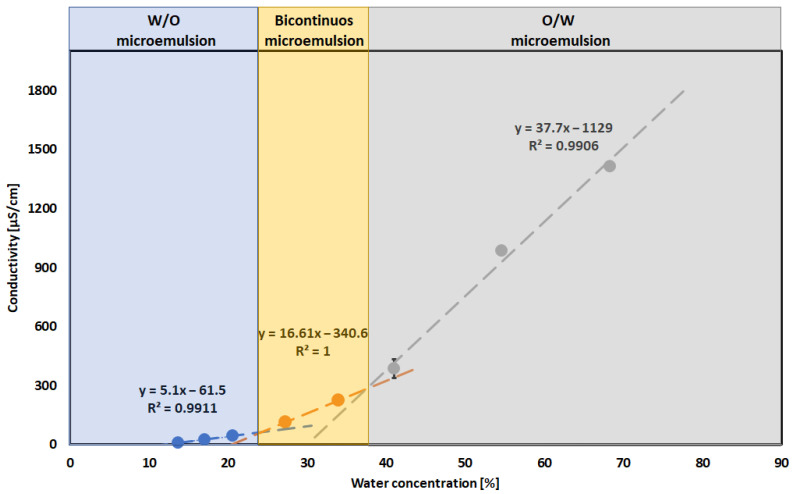
Conductivity values of the water-in-oil, bicontinuous, and oil-in-water microemulsions as a function of increasing water content in SDNE-WDS2-based formulations. The conductivity values are presented as means ± standard deviation (means ± SD); *n* = 3.

The primary microemulsion type was deduced from conductivity measurements of the continuous phase. SDNE-WDS1 exhibited negligible values, confirming its microemulsion type as W/O (Winsor system type II). Conversely, SDNE-WDS2 obtained a conductivity of 82 µs/cm, suggesting either an O/W or bicontinuous primary microemulsion. To clarify the exact nature of this Winsor system, conductivity measurements were conducted on a series of SDNE-WDS2-based formulations with increasing water content. In this experiment, a significant positive shift in the slope of the conductivity measurements indicates a transition from W/O to bicontinuous and subsequently to O/W [36]. In accordance with the conductivity study illustrated in Figure 4, three distinct slopes were identified, each indicative of a distinct Winsor system configuration (the initial slope corresponds to Type II, the middle slope to Type III, and the final slope to Type I). With a water content of 27%, SDNE-WDS2 clearly demonstrated a bicontinuous character (Winsor type III).

**Figure 5:**
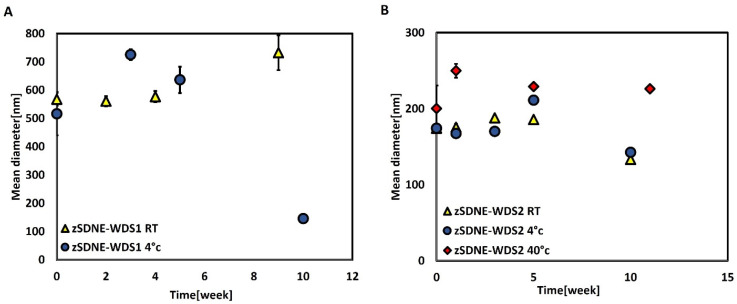
Mean droplet diameters of drug-loaded carriers over time following incubation at room temperature (RT), 4 and 40 °C. (**A**) Zanamivir-loaded SDNE-WDS1; (**B**) Zanamivir-loaded SDNE-WDS2. The results are represented as (means ± SD); *n* = 3.

**Figure 6:**
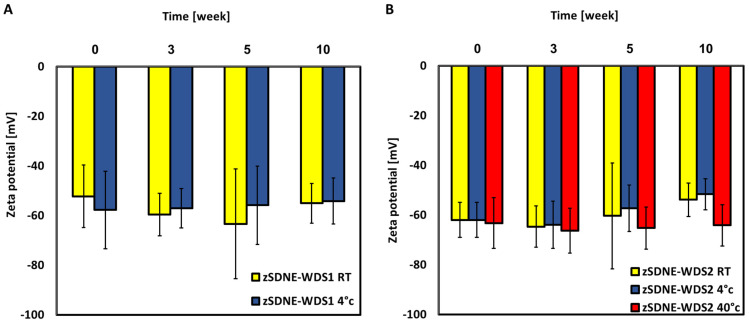
Surface charge assessment of drug-loaded carriers over time after incubation at RT, 4 and 40 °C. (**A**) Zanamivir-loaded SDNE-WDS1; (**B**) Zanamivir-loaded SDNE-WDS2. The zeta potential values are donated as (means ± SD); *n* = 3.

The mean diameters of the drug-loaded SDNE-WDSs were monitored over time, and the results are presented in Figure 5. Interestingly, SDNE-WDS1 exhibited instability at elevated temperatures. On the other hand, this formulation remained stable for at least 5 weeks at 4 °C, with slight fluctuations in averaged diameter following incubation at ambient temperature for 9 weeks (Figure 5A). Outstandingly, SDNE-WDS2 maintained long-term stability for a minimum of 10 weeks, following incubation at room temperature (RT), 4 and 40 °C (Figure 5B). The surface charge of colloidal carriers significantly influences their physicochemical stability. Overall, the zanamivir-loaded systems (excluding SDNE-WDS1 at 40 °C) consistently exhibited substantial and coherent negative zeta potential values throughout the study period, irrespective of incubation conditions (as shown in Figure 6).

### 3.2. In Vitro Release Studies

**Figure 7:**
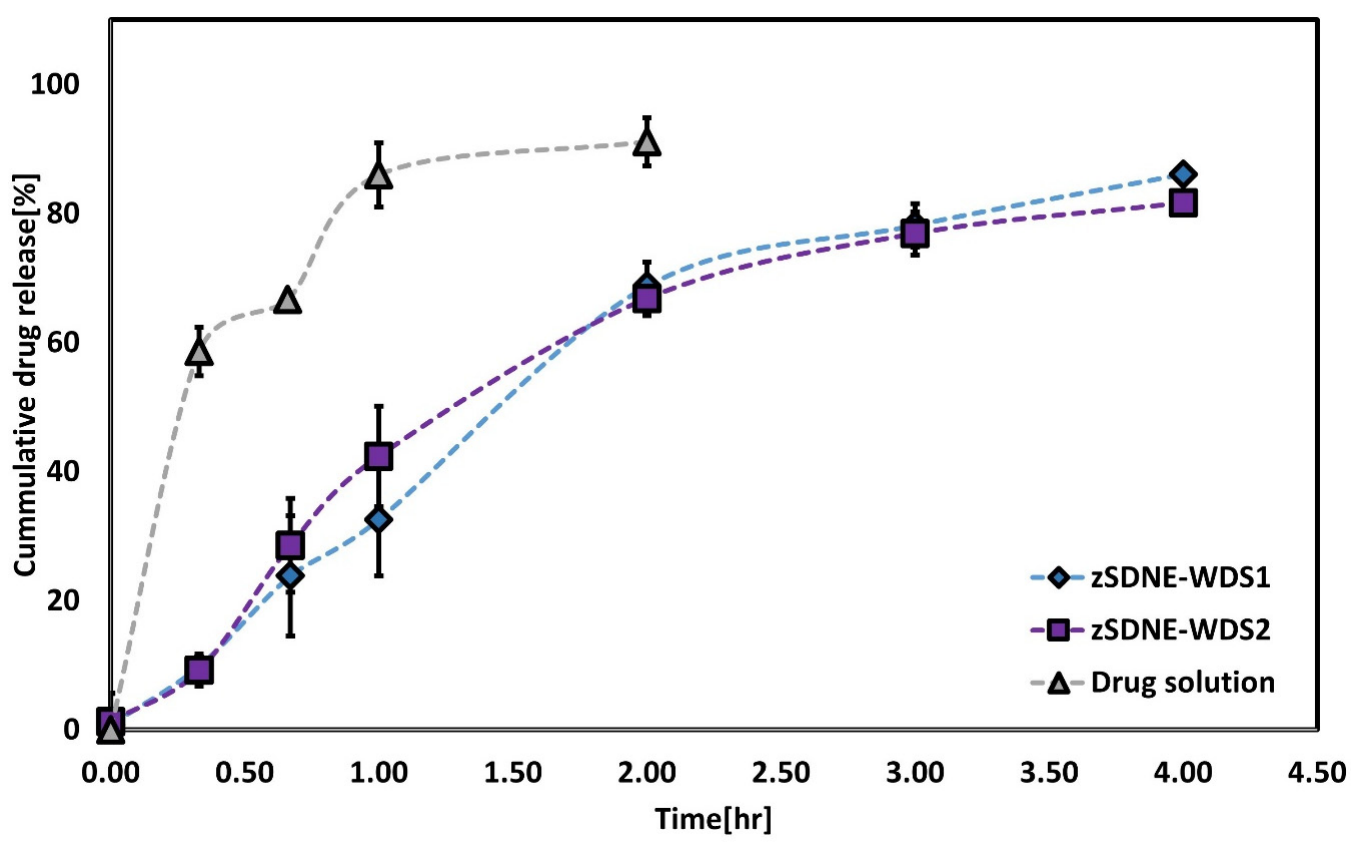
Release profiles of 400 µg/mL zanamivir from SDNE-WDS1, SDNE-WDS2, and a zanamivir solution. All formulations were tested in PBS at pH 6.8 while maintaining a temperature of 37 °C. The presented data represents the means ± SD; *n* = 9.

Figure 7 explores the results of the in vitro release experiments involving zanamivir-loaded SDNE-WDSs conducted in a phosphate-buffered saline (PBS) solution at pH 6.8 and compares them to a solution of the free drug. When examining the initial 20-minute interval, unformulated zanamivir displayed an abrupt burst release, amounting to approximately 59% of the total payload, with the entire quantity being released within the first hour. In contrast, the two distinct types of zanamivir-loaded SDNE-WDSs exhibited a more controlled release pattern. After a duration of 4 h, the cumulative release of zanamivir from SDNE-WDS1 and SDNE-WDS2 reached 86.1% and 81.6%, respectively. These findings accentuate the potential of SDNE-WDSs to modulate the release kinetics of zanamivir, providing a more sustained delivery compared to the unformulated drug solution.

### 3.3. Parallel Artificial Membrane Permeability Assay

**Figure 8:**
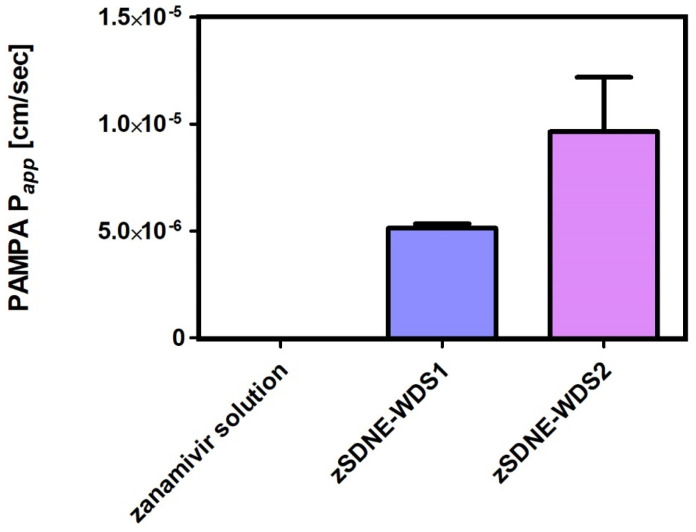
In vitro effective permeability values (P*_app_*) of 400 µg/mL zanamivir loaded SDNE-WDSs following self-emulsification, as calculated from the results obtained through the PAMPA studies conducted at pH 7.4. The results are provided as means ± SE; *n* = 4.

We conducted an in-depth investigation into the passive transport of zanamivir across an artificial membrane. Both unformulated and zanamivir-loaded double emulsions from SDNE-WDSs were subjected to this study. Following 24 h, zanamivir concentrations at the donor side were sampled, and the permeability was subsequently calculated using the acquired data (as shown in Figure 8). The results reveal the facilitated transport of encapsulated zanamivir across the artificial membrane, as demonstrated by the noticeable enhancement in the apparent permeability coefficients (P*_app_*) of this antiviral agent. ZSDNE-WDS1 and zSDNE-WDS2 exhibited significantly increased P*_app_* values, measuring at 5.14 × 10^−6^ ± 3.85 × 10^−7^ and 9.63 × 10^−6^ ± 4.41 × 10^−6^ cm/s, respectively. Remarkably, the permeability efficiency of these two formulations exhibited no marked statistical variation. These findings underline the efficacy of zanamivir-loaded SDNE-WDSs in promoting the permeation of the antiviral agent across the studied artificial membrane, potentially improving its bioavailability and therapeutic effectiveness.

### 3.4. *In Situ* Single-Pass Intestinal Perfusion Studies

**Figure 9:**
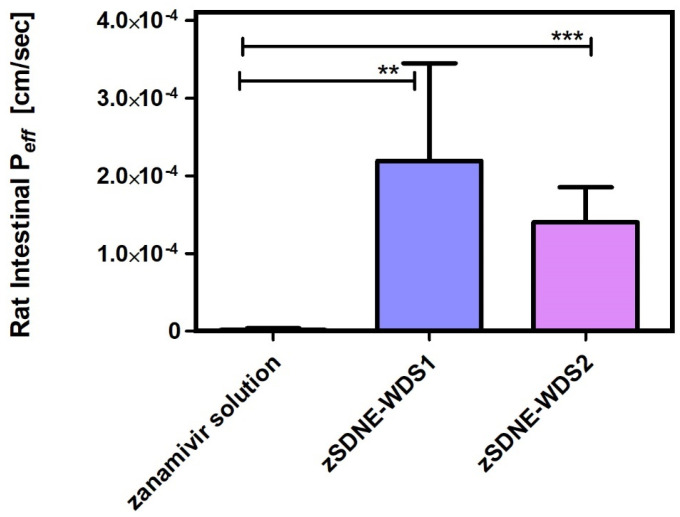
In situ effective permeability values (P*_eff_*) of 400 µg/mL zanamivir solution and zanamivir encapsulated within SDNE-WDSs, determined by perfusion of emulsions in the jejunum of rats. The results are shown as means ± SD; *n* = 6 for the zanamivir solution and *n* = 8 for SDNE-WDSs. Statistical significance is indicated as follows: ** *p* < 0.001; *** *p* < 0.0001.

We investigated the in situ effective permeability coefficients of free zanamivir, as well as when encapsulated within SDNE-WDSs by means of the single-pass intestinal perfusion rat model. This study was conducted within the proximal jejunum segment of the intestine and provided insights into the effective permeability coefficients of final nanoemulsions perfusate in comparison to those of the unformulated drug. The results presented in Figure 9 confirmed the inherently low permeability of zanamivir. Noticeably, the flux of zanamivir, when encapsulated within SDNE-WDSs, exhibited a significant increase in comparison to the free antiviral drug. The P*_eff_* values for zSDNE-WDS1 and zSDNE-WDS2 were determined to be 2.19 × 10^−4^ ± 1.26 × 10^−4^ and 1.40 × 10^−4^ ± 4.52 × 10^−5^ [cm/s], respectively. This signifies that our emulsions displayed more than a 71-fold increase in permeability compared to the free drug (*p* < 0.0004). Remarkably, our in situ model did not discover any statistical difference in the effective permeability between these two SDNE-WDS formulations.

## 4. Discussion

BCS Class III compounds are characterized by their hydrophilic nature, which translates to high aqueous solubility, but they exhibit low permeability across biological membranes. Despite their pharmacological effectiveness, their poor absorption due to low permeability becomes the limiting factor in achieving sufficient bioavailability [43]. Notably, several antiviral agents, such as adefovir, lamivudine, ribavirin, zanamivir, and zidovudine, fall into this Class III category. While these agents effectively combat some of the most prevalent viruses known to afflict humans, unfortunately, their inherently low oral bioavailability of these agents restricts their administration via the oral route.

Prior research has highlighted that the bioavailability of Class III drugs can be enhanced through their encapsulation within drug delivery systems. In numerous instances, nanocarriers have demonstrated a remarkable ability to significantly improve the efficacy of these drugs [17]. For example, the utilization of a liposomal system for tenofovir led to a 10-fold increase in drug permeability compared to the free drug in Caco-2 cultures [44]. In another study, a self-nanoemulsifying drug delivery system (SNEDDS) that incorporated adefovir dipivoxil exhibited a considerable enhancement in the oral absorption of this drug when tested in rat models [45] These findings emphasize the potential of innovative drug delivery systems to overcome the challenges posed by Class III compounds and enhance their therapeutic impact.

In this study, our strategy involved encapsulating zanamivir within innovative colloidal carriers that integrate the distinctive characteristics of self-double emulsions, microemulsions, and nanoemulsions delivery systems. Consequently, we introduced two novel self-double nanoemulsifying Winsor delivery systems, namely SDNE-WDS1 and SDNE-WDS2. These systems vary in the composition of surfactants present in the oil phase, with SDNE-WDS1 containing sorbitan laurate and SDNE-WDS2 incorporating sucrose ester. When incorporating zanamivir into these SDNE-WDSs, we achieved the formation of microemulsions through conventional emulsion preparation methods, all while avoiding the necessity for high surfactant concentrations. In typical microemulsion preparations, surfactant concentrations often surpass nearly 50%; however, our innovative approach allowed us to create microemulsions with significantly lower surfactant concentrations [46].

While the inner aqueous droplets of the primary microemulsions remained unchanged (as illustrated in Figure 3), it is important to note that the choice of oil phase emulsifiers had a significant impact on the initial Winsor system (as depicted in Figure 4). It is particularly noteworthy that sorbitan laurate promoted the formation of a W/O microemulsion, whereas sucrose stearate resulted in a Winsor type III system, as demonstrated in Figure 4. This outcome is probably attributed to the differing Hydrophilic–Lipophilic Balance (HLB) values of these emulgators, which stand at 8.7 and 11, respectively. Considering that both of our formulations had an equal overall calculated HLB, this observation is uniquely intriguing.

As depicted in Figure 4, the water content range suitable for forming bicontinuous microemulsions with SDNE-WDS2 falls within the range of 24–37%. In a study by Tamhane et al., they explored the water content range required for bicontinuous microemulsions that encapsulated a plant protease inhibitor. In their research, the water content for their Winsor type III systems was determined to be in the range of 52–58% [36]. Predominantly to date, there have not been any investigations focusing on the use of bicontinuous microemulsions for oral administration, which could open promising approaches for potential pharmaceutical applications.

The selection of different emulsifiers incorporated within the oil phase of the microemulsions also had a considerable impact on the final average sizes of the double nanoemulsions obtained (as shown in Figure 5). In particular, the mean droplet size of zSDNE-WDS2 was three times smaller than that of zSDNE-WDS1 (as shown in Figure 5). This observation possibly contributes to the significantly enhanced stability of zSDNE-WDS2, incubated in RT, 4 and 40 °C over time.

Accelerated stability studies, which involve subjecting a pharmaceutical preparation to elevated temperatures for a specific duration, can provide insights into the preparation’s shelf-life stability, often indicating a longer shelf life than the actual measured time [47]. As seen in Figure 5B, zSDNE-WDS2 demonstrated remarkable stability throughout the investigation, even under an elevated temperature environment. This extended stability can be attributed to the inherent thermodynamic stability of microemulsions, a characteristic that promotes their prolonged shelf life. The sustained long-term stability of the microemulsions developed in this study can be attributed to their small droplet size (Figure 3) and the relatively high surface charges of the final nanoemulsions (Figure 6) that collectively inhibit droplet coalescence [48].

SNEDDSs are initially composed of a single-phase oil that, upon exposure to gastric fluids and peristaltic movements, transforms into nanosized emulsion droplets. These minuscule droplets possess the capability to traverse the intestinal barrier, thereby enhancing the absorption of lipophilic drugs characterized by low bioavailability [45]. An illustrative instance involves chlorpromazine, classified as a BCS class II drug, which has been successfully loaded into SNEDDSs. The deployment of such systems has yielded a notable 2 to 6-fold increase in the area under the curve (AUC) values of chlorpromazine, surpassing the performance of the free drug in pharmacokinetic assessment [49]. However, while SNEDDSs are extensively employed for improving the bioavailability of predominantly lipophilic drugs, our investigation represents a pioneering effort. To the best of our knowledge, there has been no prior exploration into the utilization of self-emulsifying nanoemulsions for encapsulating hydrophilic drugs.

CLSM provides a powerful tool for visualizing fluorescently labeled compounds with exceptional optical microscopy resolution. In our study, we utilized rhodamine B to stain the internal aqueous phase and DPhA to stain the outer oil phase. This strategic staining allowed us to investigate whether the encapsulated hydrophilic fluorescent probe had permeated into the surrounding aqueous continuous phase or, more desirably, remained confined within the internal aqueous droplets. Figure 1 clearly indicates that rhodamine B resided within the oil droplets and exhibited no signs of leakage into the external aqueous phase. In another study, this phenomenon is further exemplified in self-double emulsions containing epigallocatechin-3-gallate (EGCG) [50].

The release profiles of zanamivir from SDNE-WDS1 and SDNE-WDS2 revealed a markedly slower release rate when compared to the rapid release observed with the free drug, as illustrated in Figure 7. Comparable release patterns have been reported in previous research involving other agents incorporated into SDEDDSs. For instance, an SDEDDS loaded with EGCG exhibited a release of approximately 30–55% of the active compound within 2 h, depending on the ratio between the oil and emulsifier used [50]. In our own investigations, both formulations exhibited nearly identical drug release profiles over time despite differences in their compositions. It is interesting to note that a similar phenomenon was observed in the release kinetics of several pidotimod SDEDDSs, where researchers explored varying phospholipid-to-cosurfactant ratios [24]. Nevertheless, Shi et al. explored Solid Lipid Nanoparticles (SLNs) as a potential formulation for zanamivir encapsulation with the aim of improving its oral delivery and enhancing its transport across the intestinal epithelial layer. Their study revealed an initial burst release effect of zanamivir from SLNs, with approximately 70% of the drug being released within the first 2 h. This burst release phenomenon is probably attributed to the presence of free drug within the system, which was not effectively encapsulated within the lipid nanoparticles [38]. The drug-loaded SLNs exhibited relatively low entrapment efficiencies for the antiviral agent, ranging from 35% to 56%, ultimately resulting in a significant amount of unformulated zanamivir being rapidly released [37,38]. A similar trend of relatively low encapsulation efficiency was observed in liposomal systems, where zanamivir entrapment ranged from 28% to 35% [51].

In contrast, previous work conducted in our laboratory demonstrated markedly higher encapsulation efficiencies for zanamivir in double emulsions, reaching levels of 96.6–98.9%, indicating a substantial degree of drug entrapment within these systems [10]. In this current study, SDNE-WDSs achieved approximately 75% drug encapsulation. This high level of drug encapsulation indicates their potential as effective carriers for zanamivir. These results stress the promise of SDNE-WDSs as a robust platform for enhancing the delivery of various hydrophilic drugs, offering potential advantages in terms of drug stability and improved intestinal permeability.

To date and to the best of our knowledge, four publications have reported on in vitro and/or in vivo attempts to enhance the oral bioavailability of zanamivir. In our previous work, we widely discussed two of these publications that attained improved bioavailability by means of prodrugs [8,33]. The remaining two papers explored zanamivir encapsulation within drug delivery systems and examined permeation coefficients across Caco-2 monolayers. In one study, liposomal zanamivir increased drug permeation by 2.2 to 3.0-fold compared to the unformulated drug [51]. In contrast, another investigation found that zanamivir penetration was actually lower than that of the control solution when encapsulated within SLNs [38]. In addition to the aforementioned studies, our recently published research elucidated the superior apparent and effective permeability coefficients observed upon the incorporation of zanamivir into double emulsion systems [10].

In the present study, the results from in vitro PAMPA studies demonstrated a significant improvement in zanamivir membrane diffusion permeability upon emulsification of SDNE-WDSs, as depicted in Figure 8. To further explore the enhancement of oral zanamivir absorption facilitated by SDNE-WDSs, we conducted in situ studies utilizing the SPIP system in rats. Notably, the transport of encapsulated molecules across the jejunum of humans is remarkably predicted using this model [7]. Our findings, illustrated in Figure 9, revealed highly promising P*_eff_* values, where SDNE-WDSs successfully converted zanamivir into a highly permeable compound, superior to the unformulated drug.

It is worth noting that the permeability values obtained in the rat model exceeded those observed in the in vitro studies. This observation is probably owing to the inherent differences between the two methods. PAMPA is a model for assessing the passive diffusion permeability across an artificial membrane. Conversely, the in situ SPIP system encompasses passive as well as active pathways involved in the permeation of a molecule through biological membranes [52,53,54].

Metoprolol offers a commonly accepted standard molecule for determining the BCS permeability classification of various compounds. In previous work, we established the permeability effective coefficient of metoprolol to be 4 × 10^−5^ cm/s, utilizing the SPIP model in rats [55]. It is particularly interesting that in the present study, under similar experimental conditions, the effective permeability values of zanamivir within SDNE-WDSs were approximately 3.5–5.5-fold higher than that of metoprolol. This outcome is particularly significant when considering the standard boundaries for low and high permeability classes. Our SDNE-WDSs have effectively masked the inherent poor permeability of zanamivir, successfully transforming this antiviral agent into a BCS class I compound characterized by high solubility and high permeability. Furthermore, in comparison to alternative carriers such as liposomes, SDNE-WDSs exhibit outstanding advantages due to their anticipated low production costs and high potential for efficient drug encapsulation [56,57].

## 5. Conclusions

The current study was undertaken to optimize the formulation of Self-Double Nanoemulsifying Winsor Delivery Systems (SDNE-WDSs) with the specific objective of augmenting the intestinal absorption of zanamivir, classified as a BCS class III compound. These SDNE-WDSs represent microemulsions that, upon emulsification, transform into colloidal carriers characterized by a high encapsulation efficacy. Results obtained from both in vitro permeability studies and in situ intestinal perfusion experiments reveal that zanamivir-loaded SDNE-WDSs exhibit enhanced intestinal membrane permeability compared to the unformulated drug. While these findings are promising, further comprehensive investigations are warranted to show pharmacokinetic analysis and to gain a deeper understanding of the precise absorption mechanisms underlying the enhanced absorption observed in the developed SDNE-WDSs.

## Data Availability

Not applicable.
